# Prognostic Factors at Diagnosis Associated With Damage Accrual in Childhood-Onset Systemic Lupus Erythematosus Patients

**DOI:** 10.3389/fped.2022.849947

**Published:** 2022-04-22

**Authors:** Ana Luisa Rodríguez-Lozano, Francisco Eduardo Rivas-Larrauri, Silvestre García-de la Puente, Daniel Alfredo Alcivar-Arteaga, Alejandro Gabriel González-Garay

**Affiliations:** ^1^Servicio de Inmunología, Instituto Nacional de Pediatría, Ciudad de México, México; Programa de Maestría y Doctorado en Ciencias Médicas, Odontológicas y de la Salud, Universidad Nacional Autónoma de México (UNAM), Ciudad de México, México; ^2^Servicio de Inmunología, Instituto Nacional de Pediatría, Ciudad de México, México; ^3^Departamento de Metodología de la Investigación, Instituto Nacional de Pediatría, Ciudad de México, México; ^4^Pediatrics Fellow, Instituto Nacional de Pediatría, Ciudad de México, México

**Keywords:** systemic lupus erythematosus, damage index, disease activity, outcome, childhood, prognosis

## Abstract

**Objectives:**

To associate prognostic factors present at diagnosis with damage accrual in childhood-onset systemic lupus erythematosus (cSLE) patients.

**Methods:**

We designed a cohort study of eligible children age 16 or younger who fulfilled the 1997 American College of Rheumatology (ACR) classification criteria for SLE. Excluded were those with previous treatment of steroids or immunosuppressants. The diagnosis date was cohort entry. We followed up on all subjects prospectively for at least 2 years. Two experts assessed the disease activity with the Systemic Lupus Erythematosus Disease Activity Index (SLEDAI) and Mexican-SLEDAI (MEX-SLEDAI) every 3–6 months. Damage was measured annually, applying Pediatric Systemic Lupus International Collaborating Clinics/American College of Rheumatology Damage Index (SDI) to their last visit. We analyzed prognostic factors by relative risks (RR) and used logistic regression to construct the clinimetric table.

**Results:**

Ninety patients with a median age of 11.8 years at diagnosis had a SLEDAI score of 15.5 (2–40) and a MEX-SLEDAI score of 12 (2–29); and of them, forty-eight children (53%) had SDI ≥ 2. The associated variables to damage (SDI ≥ 2) are as follows: neurologic disease RR 9.55 [95% CI 1.411–64.621]; vasculitis RR 2.81 [95% CI 0.991–7.973], and hemolytic anemia RR 2.09 [95% CI 1.280–3.415]. When these three features are present at diagnosis, the probability of damage ascends to 98.97%.

**Conclusion:**

At diagnosis, we identified neurologic disease, vasculitis, and hemolytic anemia as prognostic factors related to the development of damage in cSLE. Their presence should lead to a closer follow-up to reduce the likelihood of damage development.

## Introduction

Systemic lupus erythematosus (SLE) is a chronic, autoimmune, multisystem disease. Childhood-onset SLE (cSLE) develops before the eighteenth birthday and contributes to 15–20% of all patients with SLE (1). cSLE is rare in childhood, with incidence rates of 0.28–2.5 per 100,000 children/year and a prevalence of 6.3–24 per 100,000 children; variation depends on the ethnic background of the study population (2).

Damage includes any irreversible change not related to active inflammation and its causes are disease activity, side effects of medications, and co-morbid conditions (3).

Although survival has improved in the last few decades (4), patients with cSLE have higher disease activity indices than adults (5) due to being exposed to the effects of the disease for a lengthier period of time, possible relapses, the continuation of treatment, development of infections, and other pathological conditions (6) resulted in more disease damage accrual (7). In their study, Brunner et al., measured damage using the Systemic Lupus International Collaborating Clinics/American College of Rheumatology Damage Index (SDI). They compared childhood and adult cohorts after 4.5 years of follow-up and found that cSLE patients accrued mean damage of 1.70 (0–12) compared to adults who had mean damage of 0.76 (0–7) (*P* = 0.008) (8). Often considered irreversible and cumulative, damage accrual in the setting of pediatric patients might be different. Gutiérrez-Suárez et al. (3) described that some forms of damage might improve or even disappear, leading to lower scores in some pediatric patients.

Age, disease duration, antibodies to extractable nuclear antigens, renal and central nervous system affection, and more frequently, the use of steroids (9, 10) are some of the factors described in the literature associated with the development of damage in childhood. Nevertheless, damage due to disease duration and the use of steroids seems to be the natural consequence of this pathological process. Alternatively, if it can be a way to predict the outcome at the time of diagnosis, it could make clinicians more attentive to the manifestations of the disease, have a closer follow-up, and prescribe a more aggressive and earlier treatment, which will lead to lesser complications and costs. For this reason, we aimed to identify prognostic factors present at the time of diagnosis that were associated with the development of damage after at least 2 years of the diagnosis.

## Methods

The cohort study included children age 16 years or below from the Immunology Service of Instituto Nacional de Pediatría, recruited consecutively after giving consent and assent from 2012 to 2017. We decided to include this age group for a prospective follow-up of at least 2 years, as we are a pediatric center. Children must fulfill at least four of the eleven 1997 ACR classification criteria. Two specialists with more than 10 years of experience (Dr. Rivas Larrauri or Dr. Rodríguez Lozano) diagnosed and noted the diagnosis date as entry to the study. We excluded patients with concomitant immunodeficiencies, those previously treated with steroids, immunosuppressants, and biologic therapy and eliminated subjects with follow-up <2-years and those diagnosed with tuberculosis.

We designed a special sheet and collected information and disease activity at diagnosis and every 3–6 months, during the participants' visits to outpatient clinics or hospitalizations. These include a physical examination, such as weight, height, arterial tension, malar rash, photosensitivity, mucosal ulcers, palpable purpura, arthritis, serositis, and neurologic manifestations, also, laboratory workup on hemoglobin, leukocytes, lymphocytes, platelets, urinalysis, protein excretion in urine of 24-h, creatinine, complement fractions C3 and C4, Immunoglobulin G, and direct Coombs' test. Autoantibodies such as antinuclear antibodies (ANA), anti-double-stranded deoxyribonucleic acid (anti-dsDNA), anti-Smith (anti-Sm), and anti-phospholipids antibodies, namely anti-cardiolipins and lupic anticoagulants, were recorded at diagnosis and annually. We consider positive ANA test results when reported ≥1+. Our Institution report the ANA antibodies as negative or positive from 1 to 4 +. For anti-dsDNA, anti-Sm, and antiphospholipids (IgG and IgM anticardiolipin), the local reference value is >20 U/mL, and for lupic anticoagulants is >1.2 seg; we consider positive antiphospholipids if levels were above the local laboratory reference limit on at least two occasions and 3 months apart.

We took into account all types of corticosteroids the patients received. Oral prednisone, prednisolone, deflazacort, intramuscular betamethasone, pulses, or IV methylprednisolone, and IV hydrocortisone, were converted to prednisone and calculated the accumulated dose from the date of entry to the last visit. Further, we considered if any of the following neurologic diseases were present: psychosis, cerebral vascular accident (CVA), seizures, organic brain syndrome (OBS), mononeuritis, or myelitis. We scored vasculitis if palpable purpura, ulceration, tender finger nodules, periungual infarction, splinter hemorrhages, or angiogram and/or magnetic resonance images (MRI) were present. We considered hemolytic anemia if the levels of Hb were below 12 mg/dL, reticulocytes below 3%, a positive direct Coombs' tests, and/or LDH increased above 150 mg/dL. The participants were diagnosed with nephritis if proteinuria (>0.5 g/d) and/or cellular casts were present. Hypertension was defined when blood pressure was above the percentile 95th for age and height.

In this study, we measured disease activity (DA) with the Systemic Lupus Erythematosus Disease Activity Index (SLEDAI) and the Mexican–SLEDAI (MEX-SLEDAI). The main difference between the two is that MEX-SLEDAI does not determine complements nor anti-DNA antibodies, but instead takes into account hemolytic anemia, lymphopenia, and fatigue, which results in a lower costing score with similar accuracy in detecting disease activity, based on the report of Guzmán et al. (11) and our own experience (12). We evaluated DA with SLEDAI and MEX-SLEDAI (13) at diagnosis, and every 3–6 months, and considered DA if the scores were ≥4. We chose MEX-SLEDAI to report DA and its components to identify the variables associated with damage accrual in cSLE and analyzed the reliability of SLEDAI and MEX-SLEDAI, obtaining an intraclass correlation of 0.889 [0.846–0.933, *p* < 0.001]. Regarding the correlation with damage, both SLEDAI and MEX-SLEDAI have a Spearman's coefficient of 0.353, p 0.001, and 0.456, *p* < 0.001, respectively. The MEX-SLEDAI has a better correlation with damage.

Damage was assessed annually (9–15 months) with the SDI (3). We considered damage if patients had an SDI score ≥2 at the last visit (at least 2 years after diagnosis) as our outcome variable for two main reasons. Firstly, the original description of pediatric SDI (14) denotes that some forms of damage may improve or disappear, like growth velocity or delayed puberty; but a score of 2 or greater would be more defiant to recede, representing a more profound and persistent form of damage compared to an SDI score of 1. Secondly, we ran the regression analysis with Damage ≥ 1 as our dependent variable and observed that despite the model being significant (*P* < 0.001), only the hospitalization variable was statistically significant [*P* = 0.008, 95% CI 0.074–0.678]; since we aimed to look for prognostic factors, it seemed not as important. When we used an SDI score ≥2, the analysis dropped the hospitalization variable but maintained neurologic disease, vasculitis, and hemolytic anemia. We compared this last model (three variables) with the general model, and the results were not statistically significant, thus, maintaining the later model is the better option.

We (Dr. Rodríguez-Lozano or Dr. Rivas-Larrauri) verified whether the information collected in the special sheets mentioned above was correct and applied SLEDAI, MEX-SLEDAI, and SDI. To test the concordance between observers, we used a sample of 10 files. Our efforts to avoid bias included the consecutive enrollment of patients, concordance between observers, prospective follow-up, and blind and stratified analysis performed by Dr. González-Garay.

We eliminated four subjects during follow-up: one with tuberculosis infection and three with incomplete follow-ups.

Ethics Institution Board and Research Institution Board revised and approved the protocol study (Registration Number 031/2012). All participants gave their consent and assent to take part in this study. We codified the sensitive information for anonymity. No external funding was obtained to carry out this research.

## Statistical Analysis

To assess the degree of agreement between observers (Dr. Rivas-Larrauri and Dr. Rodríguez-Lozano), we conducted a concordance analysis using the Kappa index, which showed a high consistency (Kappa = 0.98).

Demographic features were reported as median (min. to max.) or frequencies (%). We used the Mann-Whitney U test to assess statistical differences where the variables were numerical and Pearson Chi-Square (or Fisher's Exact Test when appropriate) where the variables were categorical, to compare with the presence of Damage (SDI ≥ 2) vs. no Damage (SDI ≤1). We explored Spearman's correlations between accumulated steroid dose and cumulative disease activity with damage to the last visit, as they both interfere with damage accrual.

We performed a regression analysis with the variables statistically significant with Pearson Chi-Square and present at the diagnosis, along with those considered relevant to clinicians, such as gender, age, and arterial hypertension. Sample size a multivariate regression model requires 10 cases with the outcome variable (damage) for each independent variable included. We estimate that the model would incorporate 5 or 6 variables, so 50 or 60 patients with the outcome variable would be required. The final model included three variables (neurologic disease, vasculitis, and hemolytic anemia), so we considered that the sample size was sufficient. We calculated relative risks to analyze the features associated with damage (SDI ≥ 2). To construct the clinimetric table, we ran a logistic regression analysis with SDI ≥ 2 as our dependent variable.

We carried out the statistical analysis with STATA (Version 14.1, StataCorp, College Station, TX) and considered a *P* ≤ 0.05 as statistical significance.

## Results

Ninety-seven patients were diagnosed with cSLE, and upon application of selection criteria, we excluded three subjects since they had only three 1997 ACR lupus criteria. We eliminated four other patients during follow-up: one was diagnosed with tuberculosis 5 months after the SLE diagnosis, and three other patients had <2 years of follow-up. Ninety patients remained in the analysis (see Supplementary Figure 1).

### Demographic Data

Overall, there were sixty-three (70%) girls and twenty-seven (30%) boys. At diagnosis of cSLE, children had a median age of 11.8 years (2.1–16). Table 1 depicts the main clinical manifestations and laboratory findings at diagnosis, accounting for the presence of damage.

**Table 1:** Main features at diagnosis by the presence of Damage at last visit.

	**All subjects**	**Damage**	**No damage**	***P***
	***N* = 90**	**(SDI ≥2) *N* = 48**	**(SDI ≤1) *N* = 42**	
**Median (min-max)**	
Age at diagnosis (yr)	11.8 (2.1–16.1)	11.5 (2.1–16.1)	12 (3.4–16)	0.525
Time of follow-up (mo)	55.5 (24–126)	56.5 (24–126)	53.5 (24–99)	0.268
SLEDAI (score)	15.5 (2–30)	19.5 (6–40)	13 (2–30)	0.003
MEX-SLEDAI (score)	12 (2–29)	14 (5–29)	9 (2–19)	0.000
Urine Proteins (g/d)	0.6 (0–13.5)	0.8 (0–13.5)	0.1 (0–3.5)	0.097
IgG (*10^3^ mg/dl)	1.7 (0.32–5.3)	1.7 (0.32–5.3)	1.7 (0.62–3.2)	0.086
**Frequency (%)**				
Males	27 (30)	16 (59)	11 (41)	0.461
Females	63 (70)	32 (51)	31 (49)	
MEX-SLEDAI score ≥ 6	73 (81)	45 (62)	28 (38)	0.001
Neurologic Disease ^§^	17 (19)	16 (94)	1 (6)	0.000
Nephritis	58 (64)	33 (57)	25 (42)	0.362
Vasculitis ^§^	16 (18)	13 (81)	3 (19)	0.025
Hemolytic Anemia	46 (51)	32 (70)	14 (30)	0.002
Serositis	26 (29)	18 (69)	8 (31)	0.054
Hospitalization	63 (70)	41 (65)	22 (35)	0.001
ANA	90 (100)	48 (53)	42 (47)	1
Anti-DNA	55/89 (62)	29 (53)	26 (47)	0.129
Anti-phospholipids	55 (61)	26 (47)	29 (53)	1

*For numeric variables: Statistical test: Mann-Whitney U, 2-tailed. For categorical data: Statistical test: Pearson Chi-Square*,

§ *Fisher's Exact Test; 2-tailed*.

Twenty-nine (32%) patients had a background of autoimmunity, reporting 44 autoimmune diseases in relatives, the most frequent of which was rheumatoid arthritis (15 subjects, 33%), followed by systemic lupus erythematosus (13 subjects, 29%), thyroid disease (9 subjects, 20%), fibromyalgia, myasthenia gravis, and vitiligo (one subject, in each category, total 7%).

### Disease Activity at Diagnosis

Hematologic affection was the most frequent manifestation of the disease, observed in 68 participants (76%), being more prevalent among girls than boys [44 females (65%) vs. 24 males (35%); *P* = 0.06]. Mucocutaneous manifestations were the second in frequency, observed in 62 participants (69%), and alopecia was more prevalent among girls than boys [24 females (89%) vs. 3 males (11%), *P* = 0.01]. Nephritis affected 58 children (64%), with more prevalence among girls than boys [46 females (79%) vs. 12 males (21%), *P* = 0.009], but we did not find statistical significance on its components (proteinuria and casts). Concerning the impact on CNS, we did not observe gender differences. Additionally, at diagnosis, infections were observed in 31 patients, of which ten were severe and/or required admission to the intensive care unit. Seventy percent of patients (63 subjects) required hospitalization, of which 32 were for disease activity without infection.

Three patients died during follow-up, all of whom were females aged 7, 8, and 10 years at diagnosis, with long-lasting diseases (56, 54, and 76 months, respectively). All three girls developed systemic infections while receiving immunosuppressant treatment and were associated with disease activity.

### Damage Accrual

All subjects had an SDI score of zero at cohort entry and were followed up for a median of 4.6 years. We assessed one hundred percent of children for damage at first and second year, 92% for the third year, 75% for the fourth, 54% for the fifth, 30% for the sixth, 21% for the seventh, and only 9% for the eighth year. The last evaluation showed an SDI ≥ 2 in 48 children (53%). Displayed in Table 2 are the features of children at their farthest observation.

**Table 2:** Main features of children at last visit, and by Damage (SDI ≥ 2) vs. No Damage (SDI ≤ 1).

**Median (min-max)**	**All subjects**	**Damage**	**No damage**	***P***
Age, years	17.1 (6.1–18.5)	17.4 (9.9–18.6)	17.5 (8.6–18.4)	0.695
Disease duration, years	3.9 (2–9.4)	4.7 (2–10.5)	4.5 (1.8–8.2)	0.268
Added disease activity^*^	2.7 (0.2–8.1)	3.1 (0.9–8)	2 (0.2–5.8)	0.001
Cumulative steroid, g^*^	27.9 (1.2–156)	34 (3.1–156)	17.7 (1.1–54)	<0.001

*Statistical test: Mann-Whitney U, 2-tailed*.

* *Cumulative dose of steroids and added (accumulated) disease activity correlated with Damage (SDI score at last visit, Spearman coefficient 0.482, P < 0.001, 0.399 P < 0.001, respectively)*.

Growth disturbances were the most common form of damage, which accrued in 87% of children, followed by neurologic (31%) and delayed puberty (28%). Table 3 shows the main categories of SDI overall and by gender. The description of each category of the SDI score is in Supplementary Table 1.

**Table 3:** Main categories of damage (SDI) overall and by gender.

**Item**	**All subjects**	**Females**	**Males**	***P***
	***N* = 90 (%)**	***N* = 63 (%)**	***N* = 27 (%)**	
Ocular	17 (19)	8 (47)	9 (53)	0.022
Neuropsychiatric	28 (31)	18 (64)	10 (36)	0.427
Renal^§^	9 (10)	5 (56)	4 (44)	0.444
Pulmonary^§^	7 (8)	3 (43)	4 (57)	0.191
Cardiovascular^§^	5 (6)	4 (80)	1 (20)	1
Peripheral vascular^§^	8 (9)	5 (63)	3 (37)	0.692
Gastrointestinal^§^	8 (9)	4 (50)	4 (50)	0.234
Musculoskeletal^§^	18 (20)	15 (83)	3 (17)	0.251
Skin^§^	2 (2)	2 (100)	0	1
Diabetes^§^	5 (6)	4 (80)	1 (20)	1
Premature gonadal failure	5 (6)	5 (100)	–	–
Growth failure	78 (87)	57 (73)	21 (27)	0.104
Delayed puberty	25 (28)	18 (72)	7 (28)	0.797
**Items not included in SDI**	34 (38)	24 (71)	10 (29)	0.924
HTA	24 (27)	15 (63)	9 837	0.349
Depression^§^	15 (17)	12 (80)	3 (20)	0.539
Suicidal attempt^§^	3 (3)	3 (100)	0	0.551
Death^§^	3 (3)	3 (100)	0	0.551

*Statistical test: Pearson Chi-Square*,

§ *Fisher's Exact Test*.

Despite the high frequency of growth failure (87%), only 40% had short stature at their last visit; a patient with end-stage renal disease underwent renal transplantation. During follow-up, there were no patients with malignancies.

Two of the three patients who died accrued damage as both had growth disturbances and puberty delay. Other manifestations presented included retinopathy, <50% glomerular filtration rate, pericarditis, neuropathy, and muscular atrophy.

The presence of the following variables was considered as a clinical expression of damage even if SDI did not include them: depression (17%), suicidal attempt (3%), metabolic syndrome (4%), liver insufficiency (2%), chorea (2%), corneal ulceration (2%), glaucoma (2%), and hypertension (27%). One patient presented an intracardiac thrombosis; she was a female adolescent with inadequate adherence to treatment.

### Prognostic Factors to Damage

The model that best predicts damage (SDI ≥ 2) includes neurologic disease, vasculitis, and hemolytic anemia at diagnosis. Compared to the general regression model, this regression had a goodness-of-fit test (Hosmer-Lemeshow) of 2.46 (*P* = 0.652). The clinimetric table was constructed based on the regression equation, y = 1/(1+[e∧(1.084−(2.8809 _Neurologic Disease_)–(1.4491 _Vasculitis_)–(1.2746 _Hemolytic Anemia_))]). It is important to note that the probabilities of damage depend on the features present. For example, when neurologic disease, vasculitis, and hemolytic anemia are present, the probability of damage is 98.9%. When only hemolytic anemia is present, the probability is 0.5475, and when none of them are present, the probability descends to 25.2%. For details of combinations of factors, please refer to Table 4.

**Table 4:** Clinimetric assessment of Damage (SDI ≥ 2).

**Damage probabilities**	**Neurologic disease (Psychosis, CVA, Seizures, OBS, Mononeuritis, Myelitis)**	**Vasculitis (palpable purpura, MRI or Biopsy)**	**Hemolytic Anemia (Hb < 12 mg/dl, Reticulocytes > 3%, Coombs +, and/or elevated DHL)**
0.2527			
0.5475			✓
0.6023		✓	
0.8442		✓	✓
0.8577	✓		
0.9557	✓		✓
0.9643	✓	✓	
0.9897	✓	✓	✓

*Statistical test: Logistic regression*.

*CVA, cerebrovascular accident; OBS, organic brain syndrome. Different combinations of features at diagnosis resulted in the probability of damage. If none of these features are present (neurologic disease, vasculitis, and hemolytic anemia), the probability of damage is 25.2%; conversely, if all are present, the probability ascends to 98.9%*.

We calculated relative risks (RR) and 95% confidence intervals [CI 95%] for factors associated with damage (SDI ≥ 2): neurologic disease RR 9.55, [95% CI 1.411–64.621]; vasculitis RR 2.81 [95% CI 0.991–7.973], and hemolytic anemia RR 2.09 [95% CI 1.280–3.415].

## Discussion

This study aimed to identify factors present at the diagnosis of cSLE that are associated with the subsequent development of damage. We investigated the relevance of first manifestations in patients as predictive factors for damage accrual and performed a clinimetric table to help clinicians evaluate patients at the time of diagnosis. We aimed to identify three prognostic factors through multivariable analysis; neurologic disease, vasculitis, and hemolytic anemia. The clinimetric table allowed us to calculate the risk of damage for patients based on these manifestations, by themselves or in combination. The equation dropped serositis, nephritis, arthritis, fever, fatigue, infection, and hospitalization as these variables were not associated with damage. We analyzed the components of MEX-SLEDAI to calculate the impact of each manifestation.

The median age at diagnosis and proportion of males in our cohort differed from other reports. Median age (11.8 years) at diagnosis was subtly lower compared to other groups (15–19) that report patients' diagnosis at thirteen and fourteen years of age but was similar to the children in South Africa (20) and Singapore (21). The proportion of boys in this study (27 males, 30%) was broader than that of former reports (15–19) except the study of Tucker et al. (7), which showed a similar proportion of boys (13, 25%).

One-third (32%) of the patients in this study had antecedents of autoimmunity; the percentages and types of disease were similar to the study of Balci (22) and Ashournia (23) that reported 33 and 26% of autoimmunity in relatives, respectively. The most frequent types of diseases in their studies were systemic lupus erythematosus, thyroid disease, and rheumatoid arthritis, whereas, in ours, rheumatoid arthritis was more frequent, followed by systemic lupus erythematosus and thyroid disease.

Despite the survival improvement in patients with cSLE (24), damage accrual is more relevant due to its critical consequences. Damage in our cohort seemed to be more prevalent and worse than that reported by Kamphuis et al. (25), who analyzed seven studies in patients with cSLE. The damage in our cohort had a mean and standard deviation (SD) of 2.08 (1.95) and was present in 72%. These contrast the reports from elsewhere, including the international cohort reported by PRINTO (3) with a mean and standard deviation of 0.8 (1.4) in 40%, another recent study from Canada (2) shows 0.6 (1) in 39%, another in Saudi Arabia (26) shows 1.1 (1.7) in 48%, a LUMINA cohort USA (7) shows 2.3 (2.5) in 65%, another study in Lyon (27) shows 1.3 in 55%, another in Norway (28) shows 1.3 in 61%, and a former study from Canada (29) shows 2.1 (2.4) in 59%. LUMINA cohort has a large proportion of Hispanic and Black populations, which could explain the considerable damage accrued in their study. They mention multiple factors related to damage besides ethnicities, such as access to care, compliance, and socioeconomic factors.

SDI scores may increase or decrease during the evolution of the disease: when some of the items recovered as amenorrhea remits or growth velocity improves, the score may also improve. Besides, damage accrual has been related to disease duration, disease activity over time, steroid use, thrombocytopenia, presence of antiphospholipid antibodies, levels of anti-DNA, and serious infections (9, 15, 30–33).

Most of these variables take time to appear or measure and frequently develop during disease evolution; they are thus not useful to predict damage at the beginning of the disease. Nonetheless, we analyzed the relation of steroids with damage and confirmed, like in other reports (9, 30, 34), the importance of cumulative steroid doses with cumulative disease activity and damage accrual. The Spearman's correlation between damage and the cumulative dose of steroids is 0.482, *p* < 0.001. Also, the Mann-Whitney U test was significant, *p* < 0.01. The only presence of cumulative dose of steroids explains the 25% of the variability of the model to predict Damage (SDI equal to or >2) expressed by Nagelkerke R-squared of 0.257, correctly classify 65.6% of the patients, and it is statistically significant with a *P* < 0.001. However, as we mentioned earlier in the manuscript, we intend to assess the effect of the factors present at the moment of the disease diagnosis in order to help clinicians identify patients with prognostic factors at the beginning of the disease.

We calculated disease activity over time with the formula proposed by Ibañez et al. (35); for this study, x = score of MEX-SLEDAI. For initial calculation, two consecutive visits were averaged and multiplied by the number of months between these two visits, following which all the calculated areas were added up and divided by the total length of the period assessed. As expected, collinearity was observed between both cumulated steroid dose and disease activity over time with damage in the bivariate analysis. Since they are not present at the time of diagnosis, we did not include them in the assessment of prognostic factors. Owing to the nature of the study, where we aimed to identify the prognostic factors at the beginning of the disease, and we did not assess outcome variables that required time to develop, such as disease activity over time and cumulative dose of steroids, not even as confounders. The model that includes neurologic disease, vasculitis, and hemolytic anemia has a Nagelkerke R-squared 0.363, correct classification cases of 73.3%, and a *P* < 0.001, the variables neurologic disease, vasculitis, hemolytic anemia have a *P*-value of 0.008, 0.045, and 0.010, respectively; in *post hoc* analysis, if we add to the model the cumulative dose of steroids and the cumulative disease activity, then the model has a Nagelkerke R-squared 0.539, correct classification cases of 77%, and a *P* < 0.001, the variables neurologic disease, vasculitis, hemolytic anemia, cumulative dose of steroids, and cumulative disease activity have a *P*-value of 0.998, 0.199, 0.272, 0.016, and 0.551, respectively. That means that even the last model explains better the variability (Nagelkerke R-squared 0.539 vs. 0.363), the only variable that keeps its statistical significance is the cumulative dose of steroids. Besides, at the time of diagnosis, we identified and included in the model some variables that could influence the outcome, such as levels of immunoglobulin G, infection, and need for hospitalization.

Our study shares some similarities with that of Sit and Chan (15); namely, the presence of ocular and neuropsychiatric involvement as common damage manifestations and neuropsychiatric disease as a risk factor for damage development, although they considered it at any time during disease evolution. However, disease activity at diagnosis and other primary organ affection, such as hematological, renal, and serositis, were not associated with damage in their study. Conversely, the study of Pitta et al. (36) did find an association between damage (SDI equal to or >1) with neuropsychiatric disease and renal affection from the 2019 ACR/EULAR classification criteria. Our study coincides with their percentages between patients with and without damage by the presence of proteinuria >0.5 g/d, 58 and 42%, but differ in the statistical significance; the number of patients included could be an explanation, and less probable the cutoff of damage, as we replicate their cutoff (SDI equal to or >1) by proteinuria >0.5 g/d, and we obtain a *P*-value of 0.080, larger than theirs, 0.0004.

SDI does not account for depression, hypertension, metabolic syndrome, chorea, corneal ulcerations, glaucoma, and liver insufficiency in its score; hence, we decided to analyze the above mentioned as damage manifestations for the critical impact they have on the patients and their families. Depression was present in 15 subjects, of which three showed suicidal attempts. Depression, hypertension, metabolic syndrome, and liver insufficiency were present in two of the three patients who died. Chorea was present in two patients with inadequate control of the disease. Corneal ulceration and glaucoma were present in patients with long-lasting evolution; all these manifestations represent a high burden to the patients and their families. For these reasons, we agree with Holland and cols. Holland et al. (18) who suggested that SDI might not have the ability to capture damage severity properly.

Depression could be the first manifestation of cSLE (37) or be present during its evolution (38), but it is probably more common than reported in this study. Recently, Davis et al. (39) indicated in a cross-sectional investigation that 30/51 patients with cSLE reported depression when Patient Health Questionnaire-9 was applied; and was associated with non-adherence to treatment and that the worse the depression, the weaker the medication adherence. Moreover, they stated suicidal ideation in 7 patients, whereas in our cohort, we identified three subjects who intended suicide and thus merited hospitalization. Therefore, we think that depression should be considered a manifestation of damage.

There is considerable variation in the presence and severity of gastrointestinal involvement reported in the literature (40). Hepatic affection varies from almost half (41) to <1 percent (42) of the patients studied, most of whom had an equitable response to treatment and favorable outcomes. Conversely, two of our patients had an aggressive liver compromise that crucially contributed to their morbidity and mortality.

The main limitation of this study is the small sample size; about the difference in age and gender, as opposed to other Mexican studies (43, 44), we did not have a clear answer to those differences. We recruited patients consecutively, which may theoretically reduce the possibility of selection bias. Conversely, we are a reference center for autoimmune and immune-deficiencies that possibly influence the population we attend. Another limitation is the relatively brief follow-up, where to the fifth year of evaluation, only 54% of the sample was available.

At least partially, the results of this study may apply mainly to the local population; but we need to confirm these findings to extrapolate them to other populations.

This study could assist pediatric rheumatologists in identifying patients at the beginning of the disease at risk for damage, allowing them to have closer follow up with their patients and treat them more aggressively. It also may help discuss the risk of damage with patients and relatives based on objective information, supporting the arguments of the severity of the disease and its treatments, and helping them comprehend the importance of treatment adherence to limit the damage accrual.

We identified the presence of neurologic disease, vasculitis, and hemolytic anemia at diagnosis as prognostic factors related to the development of damage (SDI ≥ 2) in patients with cSLE. The clinimetric table allows us to calculate the increase in the probability of damage depending on the factors present. Pediatric rheumatologists must be aware that the occurrence of these factors at diagnosis should lead to a closer follow-up to reduce the probability of accruing damage.

## Data Availability Statement

The datasets generated and/or analyzed during the current study are available from the corresponding author upon reasonable request.

## Ethics Statement

The studies involving human participants were reviewed and approved by Comité de Ética en Investigación, officially registered at the Office for Human Research Protections of the NIH (http://ohrp.cit.nih.gov/search/search.aspx) with numbers IRB00008064 and IRB00008065. Written informed consent to participate in this study was provided by the participants' legal guardian/next of kin.

## Author Contributions

AR-L conceptualized and designed the project, also made analysis and interpretation of the data, draft the work, and provided approval and agreed for all aspect of the work. FR-L helps with the design and interpretation of the data, revised the draft critically, and provided approval and agreed for all aspect of the work. DA-A substantially contributes to data acquisition, analysis, drafting the work, and provided approval and agreed for all aspect of the work. SG-d helps with analysis interpretation, revised the draft critically, and provided approval and agreed for all aspect of the work. AG-G conceptualized and designed the project, made analysis and interpretation, revised the draft critically, and provided approval and agreed for all aspect of the work. All authors contributed to the article and approved the submitted version.

## Funding

This article was a product of the research protocol registered with the number 031/2012 approved by Instituto Nacional de Pediatría Research and Ethics Committee and received funding from the program E022 of the Fiscal Resources.

## Conflict of Interest

The authors declare that the research was conducted in the absence of any commercial or financial relationships that could be construed as a potential conflict of interest.

## Publisher's Note

All claims expressed in this article are solely those of the authors and do not necessarily represent those of their affiliated organizations, or those of the publisher, the editors and the reviewers. Any product that may be evaluated in this article, or claim that may be made by its manufacturer, is not guaranteed or endorsed by the publisher.
