# Supplementary material for: Prognostic Factors at Diagnosis Associated With Damage Accrual in Childhood-Onset Systemic Lupus Erythematosus Patients
Source: Front Pediatr. 2022 Apr 22;10:849947. doi: 10.3389/fped.2022.849947 (PMC9074833; doi:10.3389/fped.2022.849947)
Supplement: Supplementary file 1 [file Data_Sheet_1.docx]

**Supplementary Figure 1. Elegibility criteria for patients enrolled in the study.**

Identified N= 97 children with cSLE

Inclusion Criteria

N= 97

1. <17 years-old
2. At least 4 ACR lupus criteria

N=3

Patients had only three ACR lupus criteria

N=0

There were no patients excluded

Exclusion Criteria

N= 94

1. Lupus-like
2. Overlapping syndrome

N=4

1, diagnosed with tuberculosis

3, had less than two years of follow-up

Elimination Criteria

N=94

1. Loss of follow-up for more than one year
2. At least two prospective years of follow-up

Completed study

N=90
