# Supplementary material for: Prognostic Factors at Diagnosis Associated With Damage Accrual in Childhood-Onset Systemic Lupus Erythematosus Patients
Source: Front Pediatr. 2022 Apr 22;10:849947. doi: 10.3389/fped.2022.849947 (PMC9074833; doi:10.3389/fped.2022.849947)
Supplement: Supplementary file 2 [file Table_1.docx]

| **Supplementary Table 1. Subcategories description of SDI. N=90** | |
| --- | --- |
|  | **Frequencies and Percentages** |
| Ocular Disturbances |  |
| Cataracts | 5 (5) |
| Retinal changes or optic nerve atrophy | 14 (16) |
| Neuropsychiatric |  |
| Cognitive impairment | 4 (4) |
| Psychosis | 2 (2) |
| Seizures | 18 (20) |
| Cerebral Vascular Accident | 5 (6) |
| Cranial or peripheral neuropathy | 11 (12) |
| Myelitis | 2 (2) |
| Renal |  |
| Glomerular Filtration Rate <50% | 7 |
| Proteinuria > 3.5g/d | 6 |
| End-stage Renal Disease | 3 |
| Pulmonary |  |
| Pulmonary Hypertension | 3 (3) |
| Shrink lung | 1 (1) |
| Pleural fibrosis | 1 (1) |
| Pulmonary infarction | 1 (1) |
| Cardiovascular |  |
| Pericarditis | 3 (3) |
| Angina | 1 (1) |
| Mitral Insufficiency | 1 (1) |
| Peripheral vascular |  |
| Minor tissue loss (pulp space) | 1 (1) |
| Venous thrombosis | 7 (6) |
| Gastrointestinal |  |
| Resection of spleen and gallblader | 2 (2) and 3 (3) |
| Pancreatic insufficiency | 2 (2) |
| Musculoskeletal |  |
| Muscle atrophy | (18) |
| Erosive arthritis, Pathological fracture, Avascular  necrosis, and osteomyelitis | 1 (1), 1(1), 1(1), and 1(1) |
| Skin | 2 (2) |
| Diabetes | 5 (6) |
| Premature gonadal failure | 5 (6) |
| Growth failure |  |
| Cross 2 percentiles | 53 (59) |
| Delayed growth velocity | 75 (83) |
| Delayed puberty | 25 (28) |
